# 636. A Phase I Study to Evaluate the Safety, Tolerability, and Immunogenicity of a Live, Attenuated, Quadrivalent Dengue Vaccine (V181)

**DOI:** 10.1093/ofid/ofab466.833

**Published:** 2021-12-04

**Authors:** Kevin Russell, Richard E Rupp, Javier O Morales-Ramirez, Clemente Diaz-Perez, Charles P Andrews, Andrew W Lee, Tyler S Finn, Kara Cox, Amy Falk Russell, Margaret M Schaller, Jason C Martin, Donna M Hyatt, Sabrina Gozlan-Kelner, Androniki Bili, Beth-Ann Coller

**Affiliations:** 1 Merck and Co Inc., North Wales, PA; 2 University of Texas Medical Branch at Galveston, Galveston, Texas; 3 Clinical Research Puerto Rico, San Juan, Puerto Rico; 4 University of Puerto Rico School of Medicine, San Juan, Puerto Rico; 5 Diagnostics Research Group, San Antonio, Texas; 6 Merck & Co., Inc., Kenilworth, NJ; 7 Merck & Co., Inc., Kenilworth, New Jersey; 8 Merck & Co., Inc., Kenilworth, NJ, USA, Kenilworth, New Jersey

## Abstract

**Background:**

Dengue (DENV) is a mosquito-borne virus with four serotypes causing substantial morbidity in tropical and subtropical areas worldwide. A dengue vaccine that can be given to both seronegative and seropositive populations remains an important unmet medical need. V181 is an investigational live, attenuated, quadrivalent dengue vaccine.

**Methods:**

In this phase 1 double-blind, placebo-controlled study, the safety, tolerability, and immunogenicity of V181 in healthy adults were evaluated in two formulations: TV003 and TV005. TV005 has a 10-fold higher DENV2 component as compared to TV003. Two-hundred participants [~ 50% baseline flavivirus-experienced (BFE) and 50% baseline flavivirus-naive (BFN)] were randomized 2:2:1 to receive TV003, TV005, or placebo on Days 1 and 180. Immunogenicity against each of the four DENV serotypes was measured using a Virus Reduction Neutralization Test (VRNT_60_) after each vaccination and out to 1 year after the second dose.

**Results:**

There were no discontinuations due to adverse events (AEs) or vaccine-related serious AEs. The most common AEs Days 1-28 after any TV003 or TV005 vaccination were rash, headache, fatigue, and myalgia. DENV VRNT_60_ seropositivity to 3 or 4 serotypes (i.e. tri-or tetravalent) was demonstrated in 92.6% of BFN TV003 participants, 74.2% of BFN TV005 participants, and 100% of the BFE participants at 6 months postdose 1 (PD1). Vaccine viremia, a measure of vaccine infectivity, was transiently detected from all four DENV types after the first dose of TV003 and TV005. Tri- or tetravalent vaccine-viremia was detected in 63.9 % and 25.6 % of BFN TV003 and TV005 participants, respectively, PD1. Compared to baseline, robust increases in VRNT_60_ GMTs were observed after the first dose of TV003 and TV005 in both flavivirus subgroups for all DENV serotypes and minimal increases were observed PD2. GMTs in the TV003 and TV005 BFE and BFN subgroups remained above the respective baselines and placebo at 1-year PD2.

**Conclusion:**

Both formulations of V181 were generally well tolerated in healthy adults. Overall, viremia and immunogenicity were higher after TV003 as compared to TV005. These data support the continued development of the V181 TV003 formulation as a single-dose vaccine for the prevention of DENV disease.

**Disclosures:**

**Kevin Russell, MD, MTM&H**, **Merck & Co., Inc.** (Employee, Shareholder) **Richard E. Rupp, MD**, **Merck & Co., Inc.** (Research Grant or Support) **Clemente Diaz-Perez, MD**, **Merck & Co., Inc.** (Research Grant or Support) **Charles P. Andrews, MD**, **Merck & Co., Inc.** (Research Grant or Support) **Andrew W. Lee, MD**, **Merck & Co., Inc.** (Employee, Shareholder) **Tyler S. Finn, BA**, **Merck & Co., Inc.** (Employee, Shareholder) **Kara Cox, MS**, **Merck & Co., Inc.** (Employee, Shareholder) **Amy Falk Russell, MS**, **Merck & Co., Inc.** (Employee, Shareholder) **Margaret M. Schaller, BS**, **Merck & Co., Inc.** (Employee, Shareholder) **Jason C. Martin, PhD**, **Merck & Co., Inc.** (Employee, Shareholder) **Donna M. Hyatt, BA**, **Merck & Co., Inc.** (Employee, Shareholder) **Sabrina Gozlan-Kelner, MS**, **Merck & Co., Inc.** (Employee, Shareholder) **Androniki Bili, MD, MPH**, **Merck & Co., Inc.** (Employee, Shareholder) **Beth-Ann Coller, PhD**, **Merck & Co., Inc.** (Employee, Shareholder)

